# Social, health and economic impact of the COVID-19 pandemic from a European perspective

**DOI:** 10.1007/s10433-022-00744-9

**Published:** 2022-11-14

**Authors:** Thorsten Kneip, Axel Börsch-Supan, Karen Andersen-Ranberg

**Affiliations:** 1 Munich Center for the Economics of Aging (MEA), Max Planck Institute for Social Law and Social Policy, Munich, Germany; 2 Department of Economics and Business, Technical University of Munich (TUM), Munich, Germany; 3 National Bureau of Economic Research (NBER), Cambridge, MA USA; 4 Epidemiology, Biostatistics and Biodemography, Department of Public Health, Faculty of Health, University of Southern Denmark, Odense, Denmark; 5 Geriatric Research Unit, Deptartment of Clinical Research, Faculty of Health, University of Southern Denmark, Odense, Denmark

The COVID-19 pandemic and the policies to prevent the spread of SARS-CoV-2 infections vary considerably across Europe. The pandemic and the epidemic control decisions that were implemented have had major effects on the economic and social well-being of the older population: Europe is experiencing the largest recession since WWII; free movement, one of the European Union’s fundamental achievements, was interrupted and people avoided seeking medical treatment in fear of an infection.

This special section aims to contribute to a better understanding of the potential antecedents and consequences of the pandemic and investigates the non-intended effects of intervention measures on key health, economic and social outcomes across Europe. Realising this objective requires a transdisciplinary, international and longitudinal approach, such as implemented in SHARE, the Survey of Health, Ageing and Retirement in Europe. SHARE is a multidisciplinary cross-national longitudinal database of individual-level data on the health, socio-economic status and social and family networks of individuals aged 50 or over (see Börsch-Supan et al. 2013). It is *transdisciplinary* since the outcome domains—health, economic, social—are highly interconnected; *international* since the pandemic’s severity and the epidemic control decisions implemented differed across Europe and *longitudinal* since we learn the most from systematically comparing the resulting outcomes *before and after* the onset of the pandemic. The articles presented here expand on earlier research included in the EJA special section on *social, behavioural, and public health consequences of the Corona pandemic in later life*, published in June 2021 (Tesch-Römer and Lamura 2021; Arpino et al. 2021; Pan et al. 2021; Rodrigues et al. 2021) by adding (i) cross-country comparisons, (ii) a longitudinal dimension, and (iii) genuine transdisciplinarity, three core features unique to SHARE, the common database of this special section.

The main objective of SHARE is to provide high-quality data for an observatory of the health, economic and social living conditions of EU’s population at age 50 and older. The SHARE panel comprises about 380,000 interviews in the 27 EU member states, Switzerland and Israel, with observations every two years since 2004, and was kept up to date before and during the pandemic. The retention of previous respondents, including the recovery of respondents who missed a wave, is very high (about 92%, see Bergmann et al. 2019). The panel structure is enhanced by two SHARE-specific features: the *SHARELIFE histories* with retrospective questions on the entire health, income and employment history from childhood to present age (Börsch-Supan et al. 2011), and the *End-of-Life Interviews* with the next-of-kin of deceased respondents.

The SHARE data address the very age cohort that is most affected by both the pandemic and the epidemic control measures. Workers aged 50 + —about 25% of the total labour force—are especially vulnerable to job loss. Retirees are very vulnerable to wealth losses. Individuals aged 65 + are exceptionally vulnerable to the disease itself. At older ages, people are vulnerable because they often live alone or in institutions. The SHARE data allow identification of mental health and health inequalities as well as the impact of pre-existing conditions and comorbidities and precarious socio-economic conditions.

SHARE launched the Corona Survey just four months after the outbreak of the COVID-19 pandemic (Scherpenzeel et al. 2020). Its objective was to ask SHARE respondents about their experiences during the first phase of the pandemic against the background of the different lockdown measures implemented by countries. Experiences included health events such as SARS-CoV-2 infections; healthcare experiences such as lack of access, waiting lines and overcrowding; economic shocks such as unemployment or loss of income; and social shocks such as isolation or lack of help. The key advantage of these new data is their link to the SHARE panel study with its life-course information on previous health conditions and economic and social living conditions. The SHARE Corona Survey was fielded in 28 countries from June to August 2020 (*N* = 57,515). Interviews were conducted by telephone with all those who were interviewed from October 2019 to March 2020 as part of SHARE Wave 8. Hence, the data permit a person-by-person comparison of the health, economic and social situation immediately before and after the outbreak of the pandemic.

The 30-min SHARE Corona Survey has Corona-specific questions including:

- *Epidemiological questions* such as: Did you have COVID-19 symptoms? Have you been tested? Did you have COVID-19? Did you have other illnesses during the lockdown? Did your health behaviours change in response to the pandemic (smoking, drinking; wearing a mask, distancing)?
- *Questions about the quality of the healthcare system*: Did you get access to a doctor in acceptable time? Did you have to postpone an earlier planned operation? Were you treated satisfactorily in the hospital? Did you experience queues? Crowding? Unhygienic circumstances?
- *Economic questions* such as: Did you experience unemployment or short-time work? What was your loss of income? Has this loss been offset by government transfers or other means? If self-employed: What happened to your business? Were you unable to pay monthly bills? Did you need to dip into your savings?
- *Sociological questions* such as: Who were you in contact with? Who helped you? Did you help someone? Were you isolated? Did you face mental health problems?

The work in this special section was part of a major undertaking funded by the European Commission’s Directorate General for Research and Innovation under the project name “SHARE-COVID19” (Grant No. 101015924). The ongoing project’s major aims are to:

1. Identify healthcare inequalities before, during and after the pandemic and their effects across all EU Member States,
2. Understand the effects of epidemic control measures on health and health behaviours,
3. Analyse labour market implications of the lockdown, and
4. Assess the impacts of pandemic and control measures on inequality.

This special section contributes to answering these aims. It thereby advances interdisciplinary ageing research by interconnecting the most important life domains in older age—health, social environment and socio-economic conditions. The articles reflect a broad range of topics covered by SHARE at the intersection of health, social environment and behaviour. The authors come from various countries as well as different disciplines and, thus, mirror the interdisciplinary approach of investigating ageing that guides SHARE and EJA. All authors highlight implications for social policies and recommendations for strengthening European societies in times of pandemics.

The first two contributions to this special section investigate potential healthcare inequalities among older Europeans triggered by the COVID-19 pandemic and the implemented policy interventions (Aim 1). Specifically, they investigate barriers to healthcare access—forgoing healthcare because of fear of infection, having pre-scheduled care postponed, and being unable to obtain medical appointments or treatments when needed—all during the initial phase of the pandemic. In the first contribution, Smolić and colleagues combine SHARE Corona Survey and SHARE Wave 7 data with institutional and epidemic related country characteristics to examine whether, and to what extent, barriers to healthcare access vary within and across countries. They find that country differences in how the pandemic affected healthcare access can be partially explained by differences in COVID-19-related morbidity and mortality, as well as initial features of the healthcare system and the stringency of policy measures. Their findings also suggest that the sudden outbreak and rapid epidemic control measures have disproportionately crowded out the use of preventive and routine services. Economic deprivation remains a strong predictor for facing barriers to healthcare access.

The focus of the second contribution by Arnault and colleagues is the effect of prior economic vulnerability on healthcare use. They find mutually reinforcing effects of economic and health-related vulnerabilities with the adverse consequences of economic vulnerability being more pronounced among those with poorer pre-pandemic health. A cross-national comparison demonstrates that the role of economic vulnerability for reductions in healthcare access during the pandemic varies substantially across countries but does not appear to reinforce initial patterns of health inequality.

Two of the papers in this special section aim to better understand how health behaviours changed during the pandemic and in response to epidemic control measures (Aim 2). To this end, they examine which factors increase (or decrease) the likelihood of adopting precautionary behaviours (i.e. hygiene behaviours and social distancing), as prescribed or recommended by governments' containment plans. Delerue Matos and colleagues are particularly interested in the role of multimorbidity, a risk factor for severity of disease progression, intensive care need, and mortality. They show that older Europeans with multimorbidity (prior to the pandemic) were indeed more engaged in strictly precautionary behaviours than their counterparts without multimorbidity but otherwise similar demographic and socio-economic characteristics.

Bíró and colleagues add to this by investigating the time patterns of precautionary health behaviours during the summer of 2020, an easing phase of the COVID-19 pandemic in most of Europe. Their results suggest that people became less precautious over time, particularly with respect to social distancing measures. However, this was less so for those at higher risk due to chronic illness, particularly at older ages. While the finding that people that are more vulnerable were more likely to take precautionary measures to avoid infection is partially reassuring, maintaining higher levels of precautionary behaviours among the less vulnerable groups may be socially desirable, considering the positive externalities by reducing the risk of infecting (vulnerable) others.

Compliance with epidemic control measures aiming at social distancing contributes to the production of a public good: the deceleration of the pandemic. However, it comes at the (individual) cost of forgoing social interactions—with potential negative consequences, particularly for psychological well-being. The study by Atzendorf and Gruber combines SHARE data with macro-data from the Oxford COVID-19 Government Response Tracker (a database capturing policy responses around the world to COVID-19) to separate the potential medium-term effects of the pandemic from the strictness of lockdown measures on feelings of depression and loneliness among the retired European population. Their findings show that both are related to increased feelings of depression after the first lockdown, but more so for older retirees, while pandemic severity is related to increased feelings of loneliness among those living alone. At the individual level, personal contacts emerged as a key predictor of psychological well-being, while electronic contacts do not appear to be effective in mitigating the negative consequences of reduced personal contacts.

SHARE covers the 50 + European population, the younger of which are still economically active and thus at risk of being affected by crisis-induced job loss. Brugiavini and colleagues evaluate the impact of job characteristics on the probability of having experienced work interruptions and the length of such interruptions among occupationally active older Europeans (Aim 3). Based on pre-pandemic information on job characteristics (ISCO-08 3-digit job titles) from previous SHARE waves, they construct measures for remote work feasibility, required level of social interaction, as well as the essential nature of the good or service provided. The study’s key finding is that job characteristics were major determinants of the probability of labour market outcomes during the pandemic: older workers who experienced more or longer work interruption were mainly engaged in non-essential occupations either not suited to be performed remotely or involving intensive social contacts. Their results also add to a growing evidence base that the pandemic has had a greater negative impact on women’s employment compared to men’s.

In the final paper of this special section, Chłoń‑Domińczak and Holzer‑Żelażewska investigate the determinants of economic stress during the first phase of the pandemic. They consider different aspects of economic stress that may have emerged since the outbreak of the pandemic: the inability to make ends meet, receiving financial support, postponing bill payments, dipping into savings, and job loss. Their results show that the economic risks faced by older Europeans depend not only on individual characteristics but also on the characteristics of the countries in which they live. While older people in more economically developed countries on average face less difficulties making ends meet, stringent lockdown policies, including workplace closures, have increased the risk of economic stress and also reduced the chances of receiving financial assistance.

Taken together, the present selection of articles draws a broad picture of the social, health, and economic consequences of the pandemic and the control measures taken. A key message is that among the older European population, risks differ substantially across the older age groups: the young old, who are still economically active, face the risk of crisis-induced job loss and subsequent economic hardship. However, this risk differs by socio-economic factors, job profiles, and countries. Most papers find that the pandemic has increased economic, social and health inequality (Aim 4). The oldest old respond to their higher mortality risk by more pronounced precautionary behaviour, which tends to foster social isolation and to threaten their psychological well-being.

We note that all articles in this special section are based on the first SHARE Corona Survey, covering the initial phase of the pandemic. Data from the second Corona Survey, conducted during 2021 and meanwhile available to the scientific community, will further enhance our understanding of the interdependent dynamics of the pandemic and societal reactions.
